# A vacuole-like compartment concentrates a disordered calcium phase in a key coccolithophorid alga

**DOI:** 10.1038/ncomms11228

**Published:** 2016-04-14

**Authors:** Sanja Sviben, Assaf Gal, Matthew A. Hood, Luca Bertinetti, Yael Politi, Mathieu Bennet, Praveen Krishnamoorthy, Andreas Schertel, Richard Wirth, Andrea Sorrentino, Eva Pereiro, Damien Faivre, André Scheffel

**Affiliations:** 1 Max-Planck Institute of Molecular Plant Physiology, Potsdam-Golm 14476, Germany; 2 Department of Biomaterials, Max-Planck Institute of Colloids and Interfaces, Potsdam-Golm 14476, Germany; 3 Carl Zeiss Microscopy GmbH, Global Applications Support, Oberkochen 73447, Germany; 4 Department of Geomaterials, GeoForschungsZentrum Potsdam, Potsdam 14473, Germany; 5 ALBA Synchrotron Light Source, Cerdanyola del Vallés, Barcelona 08290, Spain

## Abstract

Coccoliths are calcitic particles produced inside the cells of unicellular marine algae known as coccolithophores. They are abundant components of sea-floor carbonates, and the stoichiometry of calcium to other elements in fossil coccoliths is widely used to infer past environmental conditions. Here we study cryo-preserved cells of the dominant coccolithophore *Emiliania huxleyi* using state-of-the-art nanoscale imaging and spectroscopy. We identify a compartment, distinct from the coccolith-producing compartment, filled with high concentrations of a disordered form of calcium. Co-localized with calcium are high concentrations of phosphorus and minor concentrations of other cations. The amounts of calcium stored in this reservoir seem to be dynamic and at a certain stage the compartment is in direct contact with the coccolith-producing vesicle, suggesting an active role in coccolith formation. Our findings provide insights into calcium accumulation in this important calcifying organism.

## Abstract (web summary)

Coccolithophores are unicellular marine algae that produce calcitic particles inside their cells. Here the authors study cells of the dominant coccolithophore *Emiliania huxleyi* and identify an intracellular compartment that is filled with high concentrations of a disordered form of calcium.

## Graphical abstract

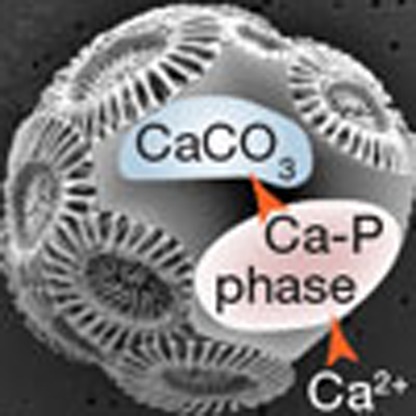

Coccolithophorid algae, a key phytoplankton group in the oceans, form elaborate arrays of minute calcite crystals known as coccoliths using Ca^2+^ and HCO_3_^−^ from the surrounding seawater^1^. This biomineralization process is of immense importance for the global cycle of carbon and other elements, as coccolithophore calcification sequesters massive deposits of CaCO_3_ into sea-floor sediments^2^,^3^. Coccolith deposits are widely used to infer past environmental conditions as environmental traces become incorporated into coccolith calcite during its formation^4^,^5^. Despite the widespread geoscientific importance of coccolithophore calcification, the cellular pathways that supply coccolith formation with the ‘building blocks' and control the elemental and isotopic composition of the final mineral remain unidentified. Uncovering these pathways will not only provide a mechanistic framework for interpreting compositional data but also the necessary foundation to address why and how coccolith calcification will be affected by the projected ocean acidification and how this process will adapt to new environmental conditions.

In the bloom-forming species *Emiliania huxleyi*, which is the best studied and dominant coccolithophore in modern oceans, coccolith formation proceeds inside an intracellular membrane system termed as ‘coccolith vesicle–reticular body'^6^. Coccoliths are formed one at a time, as fast as one per hour, and require for an average coccolith, 22.5 fmol of Ca^2+^ to be transferred into the coccolith vesicle^7^. The prevailing notion for coccolith calcite formation is that ions are transported through the cell to the site of calcite formation^8^,^9^. However, the classical, water-based preparation protocols used so far^10^,^11^,^12^ undermined the possibility of identifying highly soluble, amorphous Ca phases^13^ that, alternatively to ions, may be transported to the mineralization site as was discovered for other biomineral-forming organisms^14^.

Here we investigated *E. huxleyi* cells spectroscopically and microscopically using preparation techniques that preserve soluble, amorphous Ca phases. Using a combination of cryo-soft X-ray tomography and spectroscopy, and cryo-focused ion beam scanning electron microscopy (FIB-SEM), we visualized and characterized a highly concentrated, previously unidentified, pool of intracellular calcium and studied its corresponding ultrastructural environment. We show, using elemental analysis and live-cell staining, that polyphosphates and other elements, among them the paleomarker element Mg^5^, are co-localized with calcium, and present data that point to a possible route how calcium and other elements could be transferred to the site of mineralization.

## Results

### Speciation of intracellular calcium during coccolith formation

**Figure 1:**
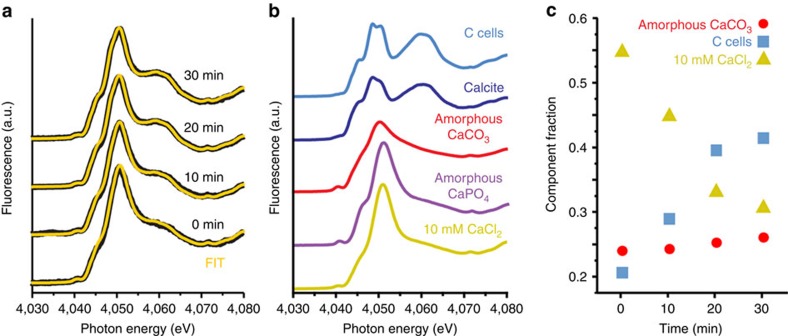
Speciation of cellular calcium during the early stages of coccolith formation in *E. huxleyi*. (**a**) Time-resolved evolution of the XANES spectra (black) of cells induced to form calcite and of the calculated fits (orange) using linear combinations of three reference standards (coccolith calcite, free calcium ions and amorphous CaCO_3_). (**b**) Ca K-edge XANES spectra of calcium reference standards and of *E. huxleyi* cells enclosed by a sphere of coccoliths (C cells). Free calcium ions were represented by 10 mM CaCl_2_ solution. The spectrum of amorphous calcium phosphate is courtesy of Diane Eichert, Elettra synchrotron, Trieste, Italy^47^. The spectrum of C cells showed the characteristic feature of calcite and was used instead of synthetic calcite for fitting the spectra of induced cells. (**c**) Relative contribution of the calcium references to the three component fits shown in **a**.

Our initial investigation of *E. huxleyi* for possible amorphous precursor phases of coccolith calcite involved X-ray absorption near-edge structure (XANES) spectroscopy. Cryogenic XANES is uniquely suited to discriminate calcium species in mixtures and played a pivotal role in the discovery of soluble inorganic phases during the formation of crystalline biominerals^15^,^16^. To follow intracellular Ca during the deposition of fresh coccolith calcite, we raised ‘calcite-free' *E. huxleyi* cells (Supplementary Fig. 1), induced calcite formation by adding Ca^2+^ to the medium and cryo-preserved cells at 10-min intervals up to 30 min, which is when calcite crystals of *status nascendi* coccoliths are detectable by cross-polarized light microscopy (Supplementary Fig. 1b). The XANES spectrum of cells before induction (0 min) had a small shoulder at 4,060 eV, which increased over time and is indicative for calcite formation (Fig. 1a). We fitted the spectra of the induced cells using linear combinations of several reference spectra (Fig. 1b). The differences between the different linear combinations were minor, even though the best fits were obtained when using amorphous calcium carbonate (ACC) in addition to CaCl_2_ solution and calcite (Fig. 1a; see Supplementary Fig. 2a,b for fits with other reference spectra). This suggests that yet unidentified amorphous Ca phases are a significant fraction of intracellular calcium at all time points (Fig. 1c).

### X-ray imaging of calcium

**Figure 2:**
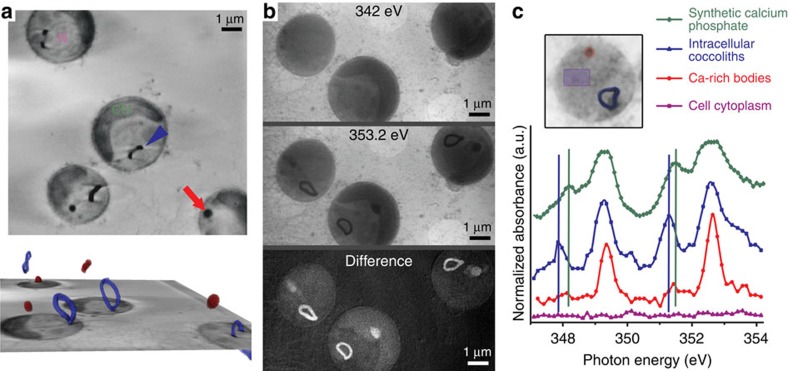
Cryo-X-ray imaging reveals concentrated calcium pools in *E. huxleyi* cells. (**a**) Two-dimensional slices from a reconstructed X-ray tomogram with (top) an immature coccolith marked by the arrowhead and a calcium-rich body marked by the arrow, and (bottom) with 3D segmentation of the calcium-rich bodies (red) and intracellular coccoliths (blue). (**b**) X-ray images recorded at an energy below the Ca L_2,3_-egde (342 eV), at the edge energy (353.2 eV) and the grey value difference between both images. (**c**) Averaged XANES spectra of the Ca L_2,3_-edge. For each spectrum, data from the relevant pixels of four cells were averaged; the inset shows the exact locations in one of these cells. Notice the difference in the position of the crystal field peak (vertical lines) between coccolith calcite and synthetic calcium phosphate. Figure is accompanied by Supplementary Movie 1.

To directly observe the putative amorphous Ca phases, we investigated cryo-preserved *E. huxleyi* cells using synchrotron soft X-ray tomography and spectromicroscopy. The minimal sample preparation procedure, consisting only of dissolving extracellular coccoliths and vitrification of cells after *de novo* coccolith formation had started, allowed the visualization of the internal organization of the cells during coccolith production in a yet unprecedented close-to-native state. Reconstruction of tilt-series images taken at an X-ray energy of 520 eV yielded three-dimensional (3D) data with a calculated spatial resolution of 52 nm, half pitch. At this energy, C-rich and Ca-rich moieties are more absorptive than aqueous solutions and therefore concentrated organic and inorganic matter appear dark, whereas the cytosol appears bright^17^,^18^. The tomograms revealed intracellular bodies with highly absorbing material, similar to the calcite of coccoliths, which were always spatially separated from the coccolith *in statu nascendi* (Fig. 2a). We probed the Ca content of these bodies by imaging cells at 342 eV, which is below the Ca L_2,3_-edge where calcium is virtually transparent, and at 353.2 eV, which is above the Ca absorption edge^19^. The difference images of the two energies revealed a distinct Ca-rich body in addition to a forming coccolith in the cells (Fig. 2b). To characterize the Ca phase of the bodies, we acquired spatially resolved Ca L_2,3_-edge XANES spectra by sequentially imaging the same field of view and varying the energy across the Ca L_2,3_-edge. Averaged spectra extracted from coccoliths *in statu nascendi* showed the characteristic spectrum of calcite (Fig. 2c). For reason explained below, we also acquired Ca L_2,3_-edge XANES spectra from synthetic, poorly crystalline calcium phosphate (Fig. 2c). The averaged spectra extracted from the Ca-rich bodies lacked significant peaks at the energy of the calcite crystal field peaks (at 347.9 and 351.3 eV) or the hydroxyapatite crystal field peaks (at 348.1 and 351.5 eV), suggesting that the body contains an amorphous calcium phase^19^,^20^,^21^,^22^. On the basis of the absorbance intensity at the Ca edge, which is proportional to the Ca content in the beam path and the volume of the Ca-rich body, the average calcium concentration in the body was determined to be ∼13.4±2.3 M.

### 3D ultrastructure of calcifying cells

**Figure 3:**
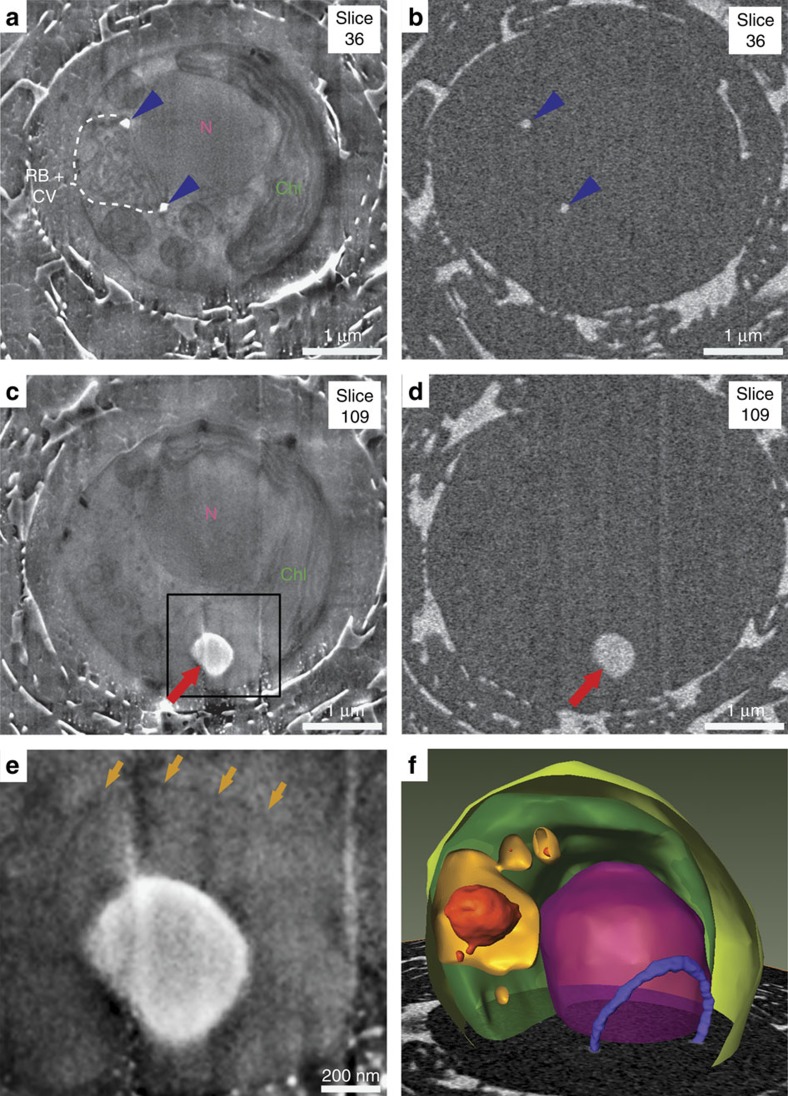
Cryo-FIB-SEM imaging of vitrified *E. huxleyi* reveals coccolith calcite and calcium-rich bodies in separate compartments. (**a**–**d**) Two slices from the same cell acquired with in-lens secondary electron detector (**a**,**c**) and energy selective backscattered electron detector (**b**,**c**) showing a cross-sectioned coccolith *in statu nascendi* (blue), the Ca-rich body (red), the coccolith vesicle (CV)–reticular body system (RB), the nucleus (N) and the chloroplast (Chl). Additional organelles are visible in the secondary electron images as is shown in Supplementary Fig. 3. (**e**) Oversampled and contrast-enhanced magnification of the area framed in **c**, illustrating the membrane that encloses the Ca-rich body (arrows). (**f**) 3D reconstruction of an *E. huxleyi* cell from a cryo-FIB-SEM image series, showing the nucleus (violet), chloroplast (dark green), plasma membrane (light green), a coccolith *in statu nascendi* (blue), Ca-rich bodies (red) and the membranes encompassing Ca-rich bodies (orange). Figure is accompanied by Supplementary Video 2.

To elucidate the ultrastructural environment of the Ca-rich body at higher resolution, we imaged cryo-fixed cells by serial FIB milling and block face SEM at cryo-condition using a Zeiss Auriga60 FIB-SEM^23^. In the energy selective backscattered (EsB) electron images, the Ca-rich bodies and coccoliths *in statu nascendi* were easily identifiable due to their calcite-like contrast (Fig. 3b,d). Image analysis, using coccolith calcite (calcium concentration is 27 M) and culture medium (calcium concentration is 10 mM) as Ca reference concentrations, yielded a concentration of 10 M Ca for the body, which is in range of the concentration determined from our X-ray tomography data set. In the in-lens secondary electron images, membrane-bound compartments were clearly visible (Fig. 3a,c). Most interestingly, a membrane was found in close proximity with the Ca-rich body, visible as thin dark line (Fig. 3c,e). Manual segmentation of this membrane in the image stacks revealed it to encompass the Ca-rich body, forming a compartment (Fig. 3f). Careful inspection of all images in the stacks found no connection between this compartment and the coccolith vesicle–reticular body system or any other visible endomembrane system. Noteworthy, the compartment was always larger than the enclosed Ca-rich body and the largest compartment had a volume of 1.5 μm^3^. If the largest compartment would be filled entirely with calcium at a concentration of 10 M, as in the Ca-rich body, it would store 15 fmol Ca, which is about two-third of the Ca required for a full coccolith.

### Compositional characterization of the Ca-rich body

**Figure 4:**
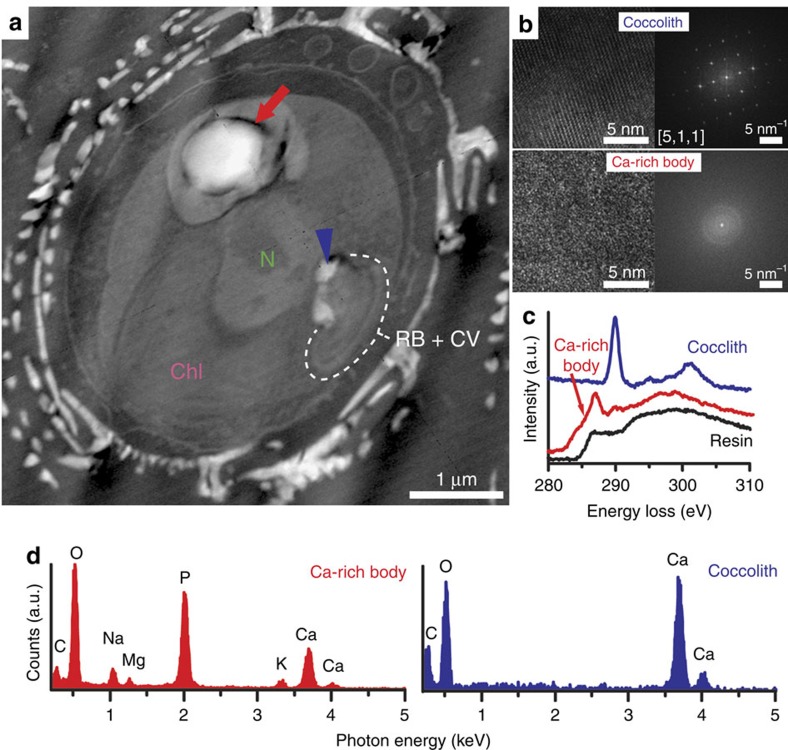
Ultrastructural and elemental microanalysis of the calcium-rich body. (**a**) HAADF-STEM image of a thin-sectioned cell showing the nucleus (N), the chloroplast (Chl), the coccolith vesicle (CV)–reticular body system (RB) (encircled by the white line), coccolith calcite (blue arrowhead) and the Ca-rich body (red arrow). Additional organelles that are visible in the HAADF-STEM images are shown in Supplementary Fig. 4. (**b**) High-resolution images and corresponding Fourier-transformed image of coccolith calcite and the Ca-rich body. (**c**) STEM-EELS spectra measured at the carbon K-edge on the Ca-rich body, coccolith calcite and embedding resin. (**d**) STEM-EDX spectra of the Ca-rich body and coccolith calcite.

We investigated the thin sections of high-pressure frozen, cryo-substituted and resin-embedded *E. huxleyi* cells using high-angle annular dark-field scanning transmission electron microscopy (HAADF-STEM) and analysed the elemental composition by energy-dispersive X-ray (EDX) spectroscopy and electron energy loss spectroscopy (EELS) at positions of interest. HAADF-STEM is suited for the imaging of the non-stained thin sections, as the image contrast is related to the atomic number of the elements in the beam path^24^. Calcium appears therefore brighter than biomolecules. We observed electron-dense Ca-rich bodies in the thin sections collected in ethylene glycol (Fig. 4a) but not in the sections collected in water. The dissolution of the bodies in water was accompanied by the dissolution of the coccolith calcite crystals, leaving an impression in the embedding resin. The same has been noticed previously by others^12^. The use of water for collecting the thin sections may therefore be the reason why the Ca-rich body was not observed in previous cytological studies in which occasionally a spacious intracellular compartment was observed but designated as chrysolaminarin vesicle^10^ or vacuole^25^ due to its highly electron transparent content, which most likely dissolved during sample preparation and could have been the Ca-rich body. When imaging the body at high magnification, no lattice fringes were observed, supporting our X-ray spectroscopy conclusion that the Ca in the body is in an amorphous phase (Fig. 4b). The carbon K-edge EELS spectra of the Ca-rich body was distinct from both coccolith calcite and the embedding resin, suggesting that the body does not contain significant amounts of calcium carbonate (Fig. 4c). Interestingly, the spectrum of the body showed a small peak at 288.2 eV, which has been previously associated with amide carbonyl bonds and suggests the presence of proteins^26^. To our surprise, the EDX analysis revealed the Ca-rich bodies to contain very high amounts of P in addition to Ca and minor amounts of other elements (Fig. 4d). Among these elements was magnesium, an important trace element in coccolith calcite, whose amount relative to calcium is used to infer past climatic conditions^5^. The excess of P over Ca in the Ca-rich body (Supplementary Table 1) suggests that Ca is bound by phosphate-rich macromolecules. Because the amount of P exceeds the amount of carbon, the most plausible candidates are not phosphorylated biomolecules but rather polyphosphates, which have been proposed to play a role in CaCO_3_ biomineralization^27^ and were discovered already in the non-calcifying relative of *E. huxleyi*, *Pavlova ennorea* sp. nov.^28^. In *E. huxleyi* polyphosphates may be synthesized by the enzyme polyphosphate polymerase (JGI ID406855)^29^.

**Figure 5:**
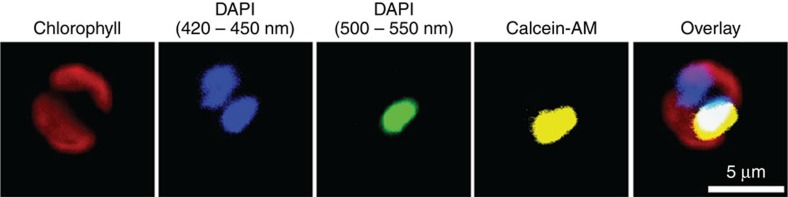
Confocal fluorescence microscopy images of live *E. huxleyi*. Cells were dual-stained with DAPI and the membrane-permeable calcium stain calcein-AM. The emitted fluorescence in the wavelength window between 420 and 450 nm originates from DAPI–DNA and DAPI–polyphosphate complexes, whereas the emission between 500 and 550 nm originates from DAPI bound to polyphosphate. The red channel shows the auto-fluorescence of chlorophyll.

We tested our polyphosphate hypothesis by live-cell confocal fluorescence microscopy using the fluorescent dye 4′,6-diamidino-2-phenylindole (DAPI), which has been widely used to stain polyphosphates^30^, and the membrane-permeable calcium stain calcein-AM. The co-localized fluorescence signals from both the dyes in a compartment distinct from the nucleus and the coccolith vesicle–reticular body system (Fig. 5) are consistent with our EDX results that strongly suggest that polyphosphate is complexing Ca in the Ca-rich compartment.

### Variability of the Ca-rich body

**Figure 6:**
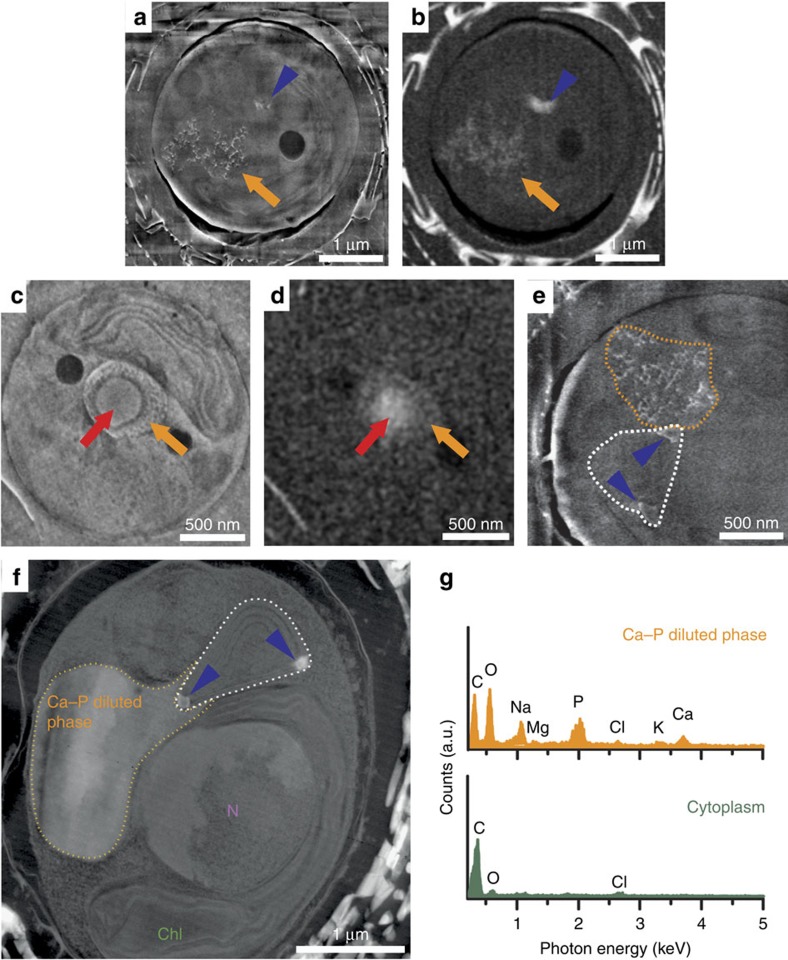
Cells without Ca-rich body contained a compartment filled with diluted concentrations of calcium and phosphorus. (**a**–**e**) Slices from cryo-FIB-SEM image series of high-pressure frozen *E. huxleyi* cells imaged in secondary electron mode (**a**,**c**,**e**) and backscattered electron mode (**b**,**d**), showing coccolith calcite (blue arrowhead), the dense Ca–P-rich body (red arrow) and a pool of diluted concentrations of Ca (orange arrow, framed orange in **e**) in close contact. The white line in **e** frames the coccolith vesicle–reticular body system. (**f**) HAADF-STEM image of a thin-sectioned cell showing the nucleus (N), the chloroplast (Chl), coccolith calcite (blue arrowhead), the coccolith vesicle–reticular body system (framed white) and the vacuole-like compartment containing Ca and P (framed orange). (**g**) EDX spectra taken from inside the compartment framed orange in **f** and of cytosol. The epoxy resin contributes to the C peak and therefore the C signal does not represent *in vivo* concentrations.

The relatively low degree of synchronization between the cells of the same culture enabled the observation of variation in the appearance of the Ca-rich body. In the cryo-FIB-SEM images of some cells, a second phase with contrast intermediate between the cytoplasm and the Ca-rich body was observed, which is most likely also Ca but at a concentration lower than in the body (Fig. 6a–c). Assigning this second phase to be a more diluted, Ca pool is supported by a similar observation that was made in the cryo-soft-X-ray images, where some Ca-rich bodies were surrounded by a scattered cloud of low concentrations of calcium (Fig. 2a). Using coccolith calcite and culture medium as Ca reference concentrations, we calculated the average Ca concentration for several ‘clouds' to be between 1 and 2 M. In accordance with these observations, we also saw in thin-sectioned cells a large compartment filled with diluted concentrations of Ca and P (Fig. 6d). The membranes delimiting the low concentrated Ca pools were seen in close contact with the coccolith vesicle–reticular body system (Fig. 6c,d). This configuration may allow the direct transfer of Ca and other trace elements found in coccolith calcite from the storage compartment into the coccolith vesicle.

## Discussion

**Figure 7:**
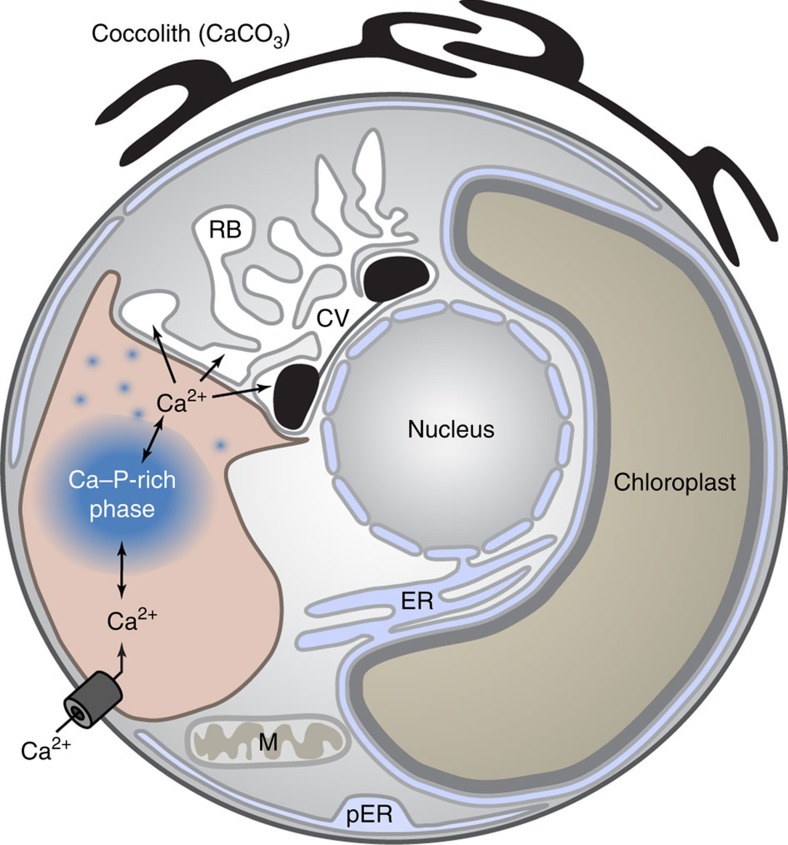
Conceptual model of the coccolith calcium pathway. Ca accumulation and calcite precipitation are spatially and temporally separated. Calcium uptake into cells involves Ca transporter. The Ca ions are concentrated by polyphosphates into a disordered phase in a compartment distinct from the coccolith vesicle–reticular body system. The disordered Ca phase is a dynamic reservoir, concentrating and dispatching Ca ions. The released Ca ions are possibly transferred into the coccolith vesicle–reticular body system by Ca transporter and/or passive diffusion driven by a high-concentration gradient in free Ca ions. Inside the coccolith vesicle the calcium is precipitated as calcite.

Our results show that *E. huxleyi* cells concentrate large amounts of Ca into a dense disordered phase in a vacuole-like compartment. Such a major internal store must be a dominant player in the cellular Ca budget and will have a pivotal role in the subcellular partitioning and allocation of Ca. The Ca phase stored in the here-discovered compartment is likely not a direct precursor phase of coccolith calcite, as (i) the Ca-rich body was never seen in direct contact with *status nascendi* coccoliths, (ii) Ca and P were not detectable in the lumen of the coccolith vesicle–reticular body system and the coccolith calcite is virtually free of P, and (iii) the calcein stain was never observed in the coccolith vesicle–reticular body system or coccolith calcite, ruling out the possibility of bulk transport from the Ca reservoir to the coccolith vesicle. The latter observation is in contrast to the situation in many other calcifying organisms where exogenously applied calcein becomes incorporated in the mineralized material^14^,^31^,^32^. Considering all the above-reported observations, the most plausible Ca transfer scenario is that Ca ions are released from their complex with polyphosphate, likely by enzymatic degradation of the polyphosphate and/or acidification of the compartment, and these are transported by Ca transporter and/or passive diffusion into the coccolith vesicle–reticular body system (Fig. 7). The large membrane surface of the reticular body has been suggested to accommodate large numbers of calcium transporters^33^. The direct contact between the membranes of both compartment systems (Fig. 6c,d), and in particular between the Ca store and the reticular body, may be advantageous as it may enable direct transfer of Ca and so bypass the cytosol where a high concentration of calcium ions could be toxic.

Ca–P-rich intracellular compartments have been reported also in multicellular CaCO_3_ mineralizing organisms^34^,^35^, but experimental challenges and the inherent complexity of these organisms have so far limited mechanistic investigation into their role in the mineral formation pathway. As simple unicellular organisms, *E. huxleyi* and other coccolithophores are attractive models for investigations on the cellular calcium budget and its use in CaCO_3_ biomineralization. The here-discovered component of the intracellular Ca pathway in *E. huxleyi* offers a new entry point into investigating the mechanistic details underlying coccolith formation and the incorporation of trace metals into coccolith calcite. The challenge ahead is to decipher the biochemical and mineralogical mechanisms underlying the formation of the Ca–P-rich phase and the reorganization of this phase in relation to the formation of calcite. Interestingly, the here-described Ca–P-rich compartment shares several characteristics with acidocalcisomes of non-calcifying organisms^36^. This makes it tempting to speculate that both the compartments are evolutionary linked. Further studies are, however, required to address this hypothesis.

*E. huxleyi* is known to thrive in waters containing low amounts of P, where other phytoplankton cannot survive^37^. The high P content of the Ca-rich body points to link between phosphate metabolism and calcification in *E. huxleyi* and may suggest that calcification is of functional significance for the ecological success of *E. huxleyi*.

Our findings for *E. huxleyi* raise the question how widespread this Ca pathway is in coccolithophores. Earlier studies revealed that in the species *Pleurochrysis carterae*, a significant amount of the calcium for synthesis of a coccolith is transported through the endomembrane system by acidic polysaccharide–Ca complexes termed coccolithosomes^38^. However, coccolithosomes have never been observed outside the genus *Pleurochrysis*, demonstrating that different Ca transport pathways evolved in coccolithophores.

The insights emerging from our study may bring the widespread interpretation of coccolith composition as proxy for seawater chemistry^5^ into a mechanistic framework and help understanding why and how calcification in coccolithophores will be affected by future climatic changes^4^,^39^, which in turn is key for developing predictive models of the future of calcification and the corresponding impact on climate.

## Methods

### Algal cultivation and induction of coccolith formation

*Emiliania huxleyi* strain AWI1516 (Alfred Wegner Institute), which produces coccoliths, was grown in artificial seawater medium Aquil, prepared according to the recipe of the National Center for Marine Algae and Microbiota, at 18 °C and a 12/12-h light/dark cycle. The concentration of nitrate in the medium was 0.2 mM and of phosphate 10 μM. The standard medium contained 10 mM CaCl_2_. Cells devoid of extracellular coccoliths were obtained by adding 1/50 volume of 0.5 M EDTA pH 8.0 to cultures and subsequent washing with fresh medium. This treatment dissolved extracellular calcite, but the calcite of coccoliths *in statu nascendi* (intracellular) remained undissolved. Cultures virtually free of coccolith calcite were obtained by repeated cultivation of EDTA-decalcified cells in modified artificial seawater medium, which contained 100 μM CaCl_2_. At 100 μM Ca^2+^, cells continued to divide, whereas coccolith formation was ceased (Supplementary Fig. 1). Calcite induction experiments were performed on calcite-free, logarithmic phase cells, 2 h after the start of the light phase. Coccolith formation was induced by the addition of CaCl_2_ to a final concentration of 10 mM. Algal cell density was measured using a Beckman Coulter Z2 particle counter.

### Light microscopy

Light microscopy was performed on an Olympus BX-51 light microscope equipped with differential interference contrast and cross-polarization optics. Confocal fluorescence microscopy was performed using an inverted laser scanning microscope (SP5, Leica, Germany) equipped with a × 63 water immersion objective (HCX PL APO CS, × 63.0, numerical aperture 1.20, Leica). Calcein-AM and chlorophyll were excited using the 488-nm line of an argon ion laser and fluorescence was recorded by photomultiplier detectors between 500 and 550 nm, and 600 and 650 nm, respectively. DAPI was imaged by two-photon excitation provided by a pulsed Ti:S laser tuned at 740 nm. The fluorescence from DAPI was recorded by photomultiplier detectors between 420–450 nm and 500–550 nm. Emission in the window 500–550 nm has been associated with DAPI–polyphosphate complexes^27^. Staining with DAPI and calcein-AM was achieved by incubating the cultures overnight with 7 and 5 μM, respectively.

### Synchrotron-based XANES

The ACC standard was prepared by adding 1/25 volume of 1 M CaCl_2_ solution to 40 mM Na_2_CO_3_ solution at room temperature. The precipitates were collected by fast vacuum filtering, dehydrated and washed with ethanol, and stored in a vacuum desiccator. Calcite was prepared by adding 1/50 volume of 5 M CaCl_2_ solution to 100 mM Na_2_CO_3_ solution. The solution was stirred for 24 h at room temperature. Calcite crystals were collected by centrifugation at 3,000*g* for 5 min, washed three times with 50 mM NH_4_HCO_3_ solutions and lyophilized. Culture aliquots before and after induction of coccolith formation were collected by centrifugation for 5 min at 4 °C. Cell pellets were washed twice with 0.7 M sorbitol (second time containing 10% dimethylsulphoxide) to remove extracellular calcium. The supernatant was poured off and the pellet was resuspended in the remaining solution. Cell suspensions were loaded into the wells (diameter 4 mm, depth 1 mm) of custom-made Cu holders, snap-frozen and stored in liquid N_2_. The XANES spectra of the Ca K-edge were acquired at the LUCIA beamline at the SOLEIL Synchrotron Light Source (Saint-Aubin, France) by scanning the X-ray beam energy from 4,000 to 4,080 eV in 1.0 eV steps before the pre-edge and 0.2 eV steps after the pre-edge. All samples were measured under vacuum and at cryo-conditions. The synchrotron ring energy was 2.75 GeV and the current was up to 400 mA. The X-ray energy was selected by a double-crystal Si(111) monochromator, which was calibrated using Ti foil and setting the edge at 4,966 eV. The beam size on the sample was ∼1.5 × 1.5 mm^2^. Spectra of algal samples and CaCl_2_ solutions were collected on snap-frozen material, whereas the spectra for ACC and calcite were collected from powdered samples mounted on sticky tape. To improve the signal-to-noise ratios, all spectra were based on an average of three to five scans. Baseline subtraction, normalization, and data processing and analysis were performed using the Athena software package^40^. The edge was taken as the maximum of the third peak of the first derivative of the XANES data. Normalization was performed using a linear pre-edge function between 25 and 13 eV below *E*_o_ and a quadratic polynomial for the post-edge between 26 and 90 eV above *E*_0_. Linear combination fitting was performed on all cell spectra using three calcium standards, 10 mM CaCl_2_, ACC and coccolith-bearing cells, C cells, whose spectrum was identical to crystalline calcite. The 10 mM CaCl_2_ solution represented free calcium ions.

### Cryo-FIB milling and SEM imaging (cryo-FIB-SEM)

*E. huxleyi* cells were collected by centrifugation, high-pressure frozen in a Leica HPM 100 apparatus using type-B sample holders (Ted Pella Inc., USA) and stored in liquid N_2_. Mounting and sputter coating, and cryo-FIB-SEM imaging were performed as described previously^23^. At liquid N_2_ temperature, the samples were mounted on a cryo-sample holder and transferred to the Leica SCD500 cryo-sputter coater, using the VCT100 cryo-transfer shuttle system (Leica Microsystems, Austria). The samples were sputter coated with a 6-nm platinum layer at 0.06 nm s^−1^ and then transferred to the Zeiss Auriga60 FIB SEM microscope (Carl Zeiss Microscopy GmbH, Germany), using the VCT100 shuttle system. Inside the microscope, a viewing channel for SEM imaging was directly milled into the sample surface using the 16-nA FIB current at 30 kV acceleration voltage (16-nA FIB probe at 30 kV). This initial coarse cross-section was fine polished using the 2-nA FIB probe at 30 kV. In a fully automated process, data cubes of serial SEM images were acquired. In a serial manner, a thin slice of material was removed by FIB milling followed by SEM imaging of the freshly exposed block face. For FIB slicing, the 240-pA FIB probe at 30 kV was used. After each milling step (step thickness 15 nm), the specimen was imaged by SEM at 2.53 kV acceleration voltage using the 10-μm aperture. For each slice secondary electron and backscattered electron images were acquired simultaneously using the in-lens secondary electron and the EsB detector (EsB grid 1,500 V). The image resolution was 2,048 × 1,536 pixels, lateral image pixel size was 5 nm and the slice thickness was 15 nm. Images were recorded using line averaging (*N*=51) and a dwell time of 200 ns. The cycle time for recording an individual image was 36.1 s. The milling time for removing each slice was 13.4 s. The image series used to reconstruct the cell shown in Fig. 2d consisted of 200 individual slices, representing a volume of 10.2 × 7.6 × 3 μm. Throughout the preparation and data acquisition process, the temperature was never higher than −150 °C. Image processing and segmentation of data cubes were performed as follows: secondary electron images were Fourier filtered to remove the vertical stripes arising from the water fall effect by FIB milling and aligned automatically with the ‘Linear Stack Alignment with SIFT' plugin of ImageJ (http://rsbweb.nih.gov/ij/index.html), allowing only for translations. The same translation applied to each image of the secondary electron stack was then applied to the corresponding image of the EsB stack. Segmentation of structures of interest was performed using Amira 3D (FEI, USA). Organelles were segmented manually from secondary electron image stacks using the lasso tool. The Ca-rich bodies were segmented automatically from EsB image stacks using the Magic Wand tool. Surfaces were generated from segmented structures and smoothed. The MaterialStatistic module was used to calculate volumes and average grey scale intensities (EsB image stack only) of segmented structures. The grey scale intensities in the EsB image stacks were used for the quantification of calcium within the Ca-rich bodies. The grey scale intensities were calibrated against the backscattered coefficient (*η*) of materials with known composition (internal standards). The calibration line was used to estimate *η* at positions of interest. From *η*, the concentration of calcium was retrieved. In detail, lipid bodies, artificial seawater medium and external coccoliths were used as internal standards. The effective backscattered coefficient for every internal standard, *η*_eff_, was estimated using the mixture rule: *η*_eff_=∑*η*_i_ × *c*_*i*_, where *c*_*i*_ is the weight fraction of the *i*th atom and *η*_*i*_ the corresponding atomic backscattered coefficient. *η*_*i*_ was calculated for each atom using the empirical Heinrich's formula under the assumption that *η* for low atomic weight elements (*Z*<20) does not change significantly with the primary beam energy^41^. Mass weight fractions for every atom (that is, the *c*_*i*_ coefficients) were calculated under the assumption that (1) lipid bodies are constituted of linear hydrocarbons (largely CH_2_), (2) the *c*_*i*_'s of the extracellular solution are known from the composition of the seawater medium, (3) external coccoliths are made of pure calcite. From the calibration line (EsB intensity versus *η*_eff_), the effective backscattered coefficient for the Ca-rich body was derived and, assuming that the Ca-rich body is a concentrated solution of electrolytes with a Ca:P atomic ratio of 1:2 (Supplementary table 1), the mass fraction of calcium ions was calculated. From this, the molarity of calcium inside the Ca-rich bodies was approximated by 1/*ρ*_s_=*c*_w_/*ρ*_w_+∑*c*_*i*_/*ρ*_*i*_, where *c*_w_ and *ρ*_w_ are the weight fractions and the density of water, respectively, and *c*_*i*_ is the weight fractions of the electrolytes and *ρ*_*i*_ their densities calculated from their atomic radii.

### Analytical scanning transmission electron microscopy

Cells were high-pressure frozen with 1-hexadecane (Sigma-Aldrich) as cryoprotectant and freeze-substituted with 2% (w/v) osmium tetroxide (Electron Microscopy Sciences) and 8% (v/v) 2,2-dimethoxypropane (Sigma-Aldrich) in acetone for 5 days at −85 °C. Samples were warmed-up to room temperature over 2 days and embedded in Spurr's resin over 4 days. Resulting resin blocks were sectioned to ∼130 nm using a Leica UC-6 microtome. Thin sections were collected in pre-cooled ethylene glycol and mounted on copper grids. Dark-field electron imaging, EELS and EDX spectroscopy were performed on a Tecnai G2 F20 X-Twin microscope equipped with a field-emission electron source (operated at 200 kV), a Fishione HAADF detector at 330 mm camera length (diffraction contrast+Z contrast) for image acquisition in the STEM mode, a Gatan Tridiem imaging filter for acquisition of energy-filtered images and an EDAX Genesis X-ray analyser.

### Soft X-ray cryo-tomography and spectromicroscopy

Cells were EDTA-decalcified and allowed to resume coccolith formation in normal medium for 3 h before collection by centrifugation. Cells in 3-μl medium and 1 μl of a 100-nm latex bead solution (2.5%, Sigma-Aldrich) were placed together on top of Quantifoil R 2/2 holey film copper transmission electron microscopy grids (Quantifoil, Germany). The beads served as fiducial markers for the tomographic reconstruction. The sample was placed in a humidified chamber, manually blotted and plunge-frozen in liquid ethane using a Cryoplunge 3 system (Gatan Inc., USA). The frozen samples were kept at cryogenic conditions throughout shipment, mounting and imaging. Hydroxyapatite was synthesized by mixing 0.1 M CaCl_2_ solution with 0.1 M Na_2_HPO_4_ solution in a similar protocol to the synthesis of the calcium carbonate samples for XANES analysis. X-ray imaging was performed at the MISTRAL beamline (ALBA Synchrotron, Barcelona, Spain)^42^,^43^. Initially, a tilt series at 520 eV X-ray energy was collected to allow a 3D volume reconstruction of cells and their internal structures. At this energy, carbon-rich, as well as Ca-rich, molecules have an intense contrast against the water-rich cytoplasm. The tilt series consisted of 121–131 images taken at 1° degree intervals. Exposure time was 1–2 s to maximize signal-to-noise level and minimize radiation damage. The data sets were acquired using a zone plate objective lens with an outermost zone width of Δ*r*_n_=40 nm. The effective pixel size in the images was 11.8 nm. The projection images were normalized using flat-field images of 1-s exposure (incoming flux delivered by the capillary condenser lens) and corrected for changes in the electron beam current. Alignment and reconstruction of the tilt series were carried out with IMOD^44^. For tomogram reconstruction, the simultaneous iterative reconstruction technique^45^ was used. The resolution of the tomograms was calculated by the fourier shell correlation (FSC_e/o_) criterion^46^ using a threshold of 0.25. The visualization and segmentation of the final volumes were carried out using the software Amira 3D (FEI, USA). Energy scan series around the Ca L_2,3_-edge followed the tilt series. In an energy scan, the same field of view was repeatedly imaged under changing X-ray energies starting at 347.7 eV and going up to 354.7 eV in 0.15 eV steps, which is approximately half the energy resolution of the beamline for the used slits configuration. The objective zone plate lens and the charge-coupled device detector positions were automatically adjusted to maintain focus and constant magnification. Intracellular coccoliths were used as internal standard for the calibration of the absolute value of the energy. The extraction of a XANES spectrum for a specific area in the image was carried out by plotting the averaged intensity of pixels in this area, as a function of the energy at which the image was taken. First, the images of the different energies were aligned. For the spectrum of intracellular coccoliths, all pixels being part of a coccolith were manually marked in four cells and the intensity value plotted corresponded to the averaged intensity of all pixels. Analogously, the spectrum of Ca-rich bodies and the cytoplasm were obtained. Calcium localization in the samples was carried out by subtracting an image taken at 342 eV (before the Ca L-edge) from an image taken at 353.2 eV (at the peak of the second white line of the Ca L-edge). The contrast in the difference image is dominated by areas containing substantial Ca content. To exclude a possible radiation damage that can affect the spectroscopic features of the Ca atoms, a second energy scan was conducted after the first one. The identical spectra in both the scans confirmed that the irradiation did not affect the Ca local structure. The combination of Ca absorption at a specific location and the 3D data on the geometry of the Ca-rich bodies enabled the estimation of Ca concentration from the acquired data. The pixel intensities in the difference images, acquired using energy-resolved X-ray microscopy, give a semi-quantitative estimation of the content of Ca atoms in the X-ray path through this location. To estimate the concentration from the amount of calcium present in the X-ray path, the length of the Ca-rich volume at the relevant orientation was measured in the 3D reconstructed data. Intracellular coccoliths were used as internal standards to calibrate the Ca concentration in calcite which is 27 M. On four cells, the Ca concentration of calcite was measured with 13.7% s.d.

## Additional information

**How to cite this article:** Sviben, S. *et al*. A vacuole-like compartment concentrates a disordered calcium phase in a key coccolithophorid alga. *Nat. Commun.* 7:11228 doi: 10.1038/ncomms11228 (2016).

## Supplementary Material

Supplementary Information Supplementary Figures 1-4 and Supplementary Table 1

Supplementary Movie 1 Three-dimensional reconstruction of *E. huxleyi* cells from a tomogram obtained by X-ray cryotomography. Tour through the tomographic volume of four vitrified cells, followed by 3D segmentation of the chloroplast (dark green), nucleus (violet), a coccolith *in statu nascendi* (blue) and the Ca-rich body (red). The coccolith *in statu nascendi* is closely associated with the nucleus.

Supplementary Movie 2 Three-dimensional reconstruction of an *E. huxleyi* cell from a cryo FIB-SEM image series. Sequential images through a vitrified cell acquired with the energy selective backscattered detector, followed by sequential images through the same cell acquired with the In-lens secondary electron detector, followed by revealing the 3D segmentation of the plasma membrane (light green), the chloroplast (dark green), nucleus (violet), a coccolith *in statu nascendi* (blue), Ca-rich bodies (red) and the membranes (orange) encompassing the bodies. As the contrast in backscattered imaging correlates with the mean atomic number of the material, mature coccoliths (part of coccosphere) and the coccolith *in statu nascendi* (intracellular) as well as the Ca-rich body appear bright. It corresponds to Figure 3.
