# Supplementary material for: A vacuole-like compartment concentrates a disordered calcium phase in a key coccolithophorid alga
Source: Nat Commun. 2016 Apr 14;7:11228. doi: 10.1038/ncomms11228 (PMC4834641; doi:10.1038/ncomms11228)
Supplement: Supplementary Information — Supplementary Figures 1-4 and Supplementary Table 1 [file ncomms11228-s1.pdf]

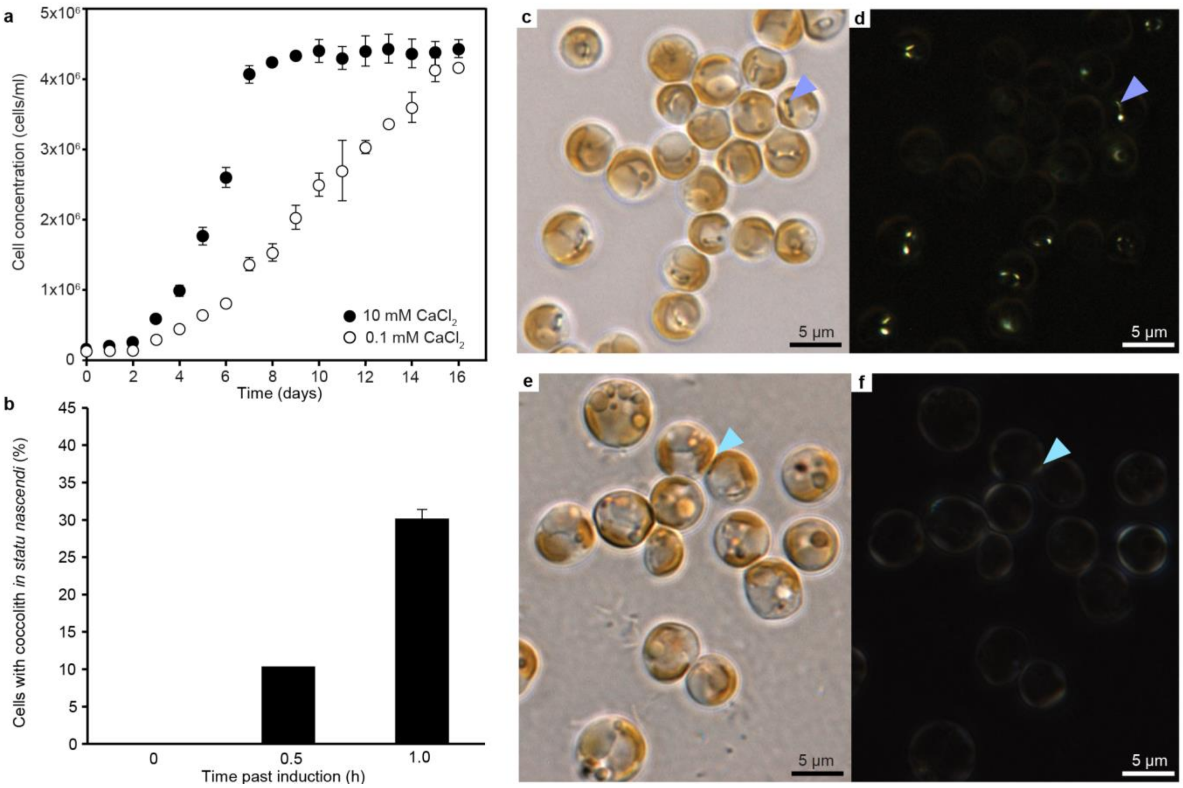


**Supplementary Figure 1 | Turning coccolith formation 'on' and 'off'.** (**a**) Growth behavior of *Emiliania huxleyi* in standard (10 mM $CaCl_2$) and calcium-depleted (0.1 mM $CaCl_2$) artificial seawater. The values are the mean of triplicate samples, and the bar indicates the standard error. (**b**) Plot showing the proportion of cells with an intracellular coccolith at different time points after induction of coccolith formation in calcium-depleted *E. huxleyi*. Bar graphs showing mean±s.d. of triplicate experiments with >500 cells in each experiment analyzed. (**c,d**) Light micrographs of EDTA-decalcified *E. huxleyi* cells. Arrowhead points to an intracellular coccolith and indicate the same position in the bright-field (**c**) and the cross-polarized light (**d**) image. Note that in cross-polarized light imaging contrast is related to birefringent material such as calcite. (**e**,**f**) Light micrographs of *E. huxleyi* cells grown for two weeks in calcium-depleted medium, which turns coccolith formation 'off'. Arrowhead indicates the same position in the bright-field (**e**) and the cross-polarized light (**f**) image.

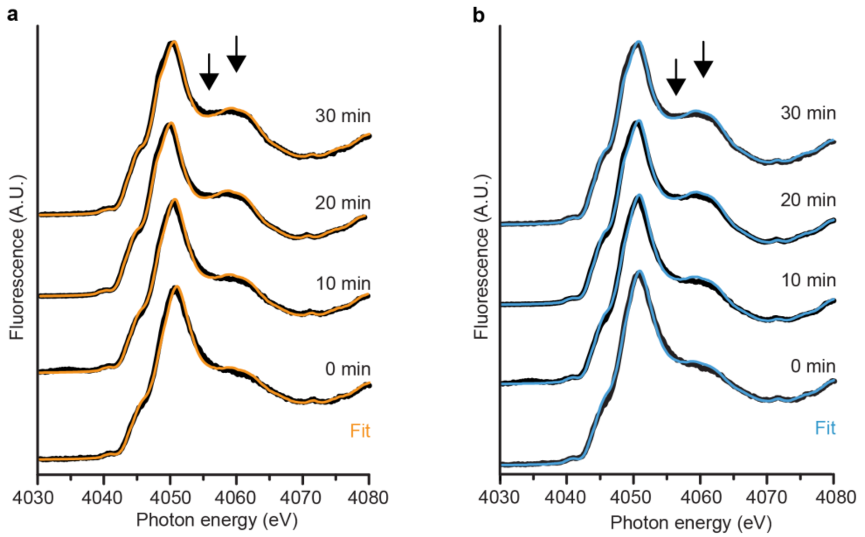


**Supplementary Figure 2 | Speciation of cellular calcium during the early stages of coccolith formation in *E. huxleyi*.** Time-resolved evolution of the XANES spectra (black) of cells induced to form calcite and of the calculated fits using linear combinations of (**a**) the two reference standards (orange), coccolith calcite and free calcium ions, and (**b**) the three reference standards (blue), coccolith calcite, free calcium ions, and amorphous calcium phosphate. Arrows point to regions of highest divergence between fit and measured data.

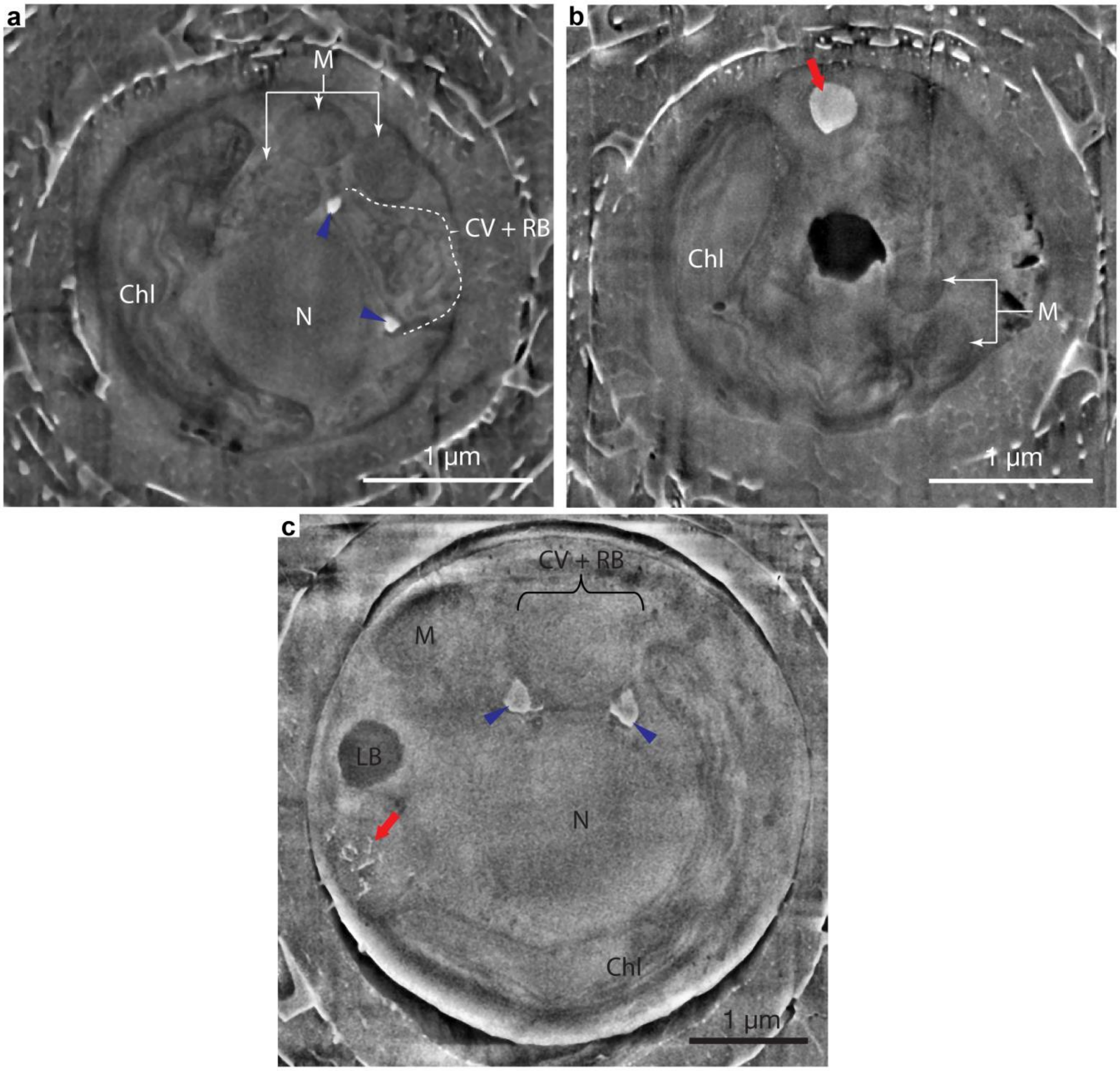


**Supplementary Figure 3 | Cryo FIB SEM imaging of vitrified *E. huxleyi* cells.** 2D-slices from two cells ((a,b) cell 1; (c) cell 2) acquired with the In-lens secondary electron detector showing a cross-section of the coccolith *in statu nascendi* (blue arrowheads), a cross-section of the coccolith vesicle (CV) – reticular body (RB), a pool of diluted concentrations of calcium (red arrow), a lipid body (LB), nucleus (N), chloroplast (Chl), and mitochondria (M).

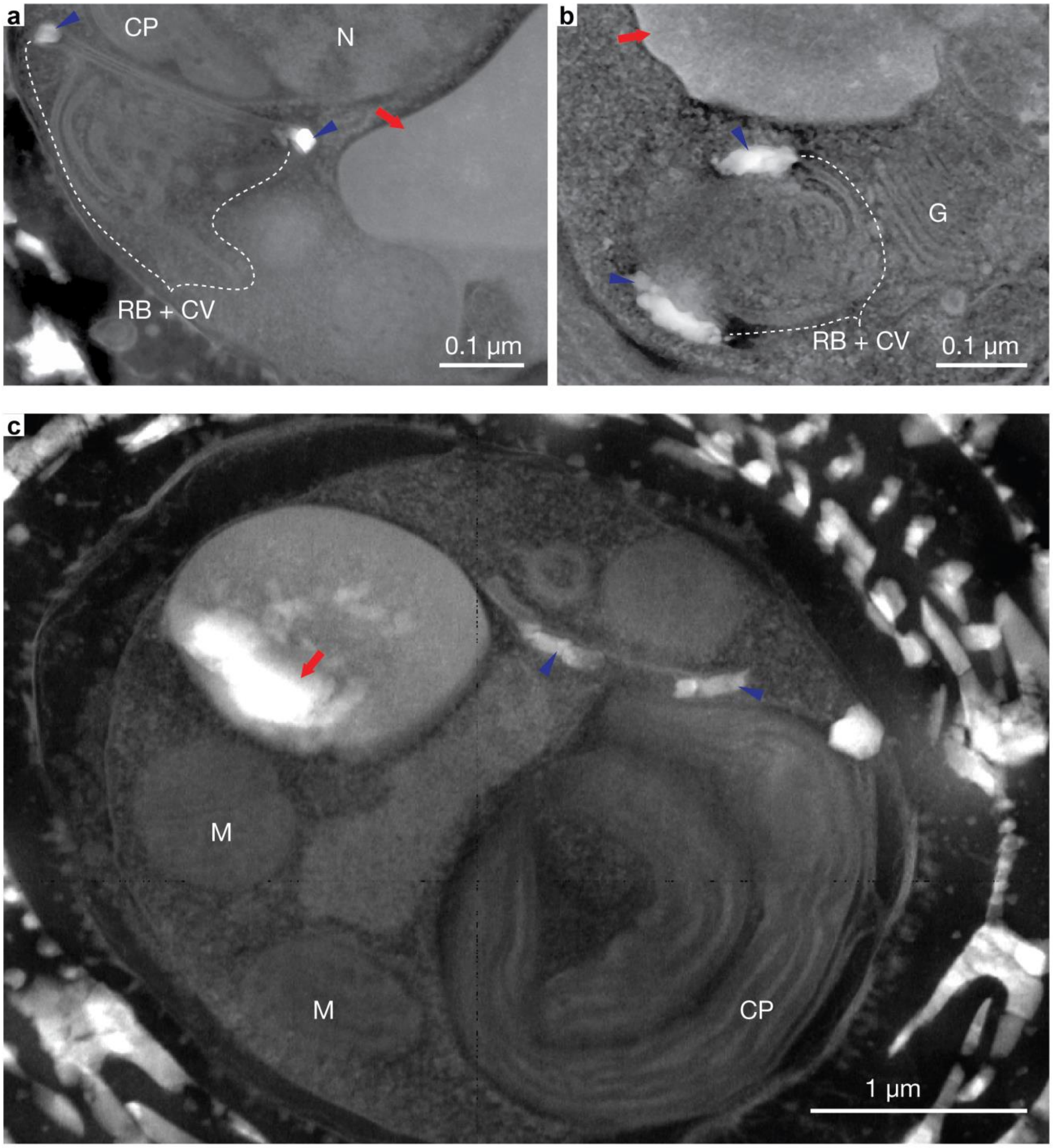


**Supplementary Figure 4 | Ultrastructural features in unstained cell sections of *E. huxleyi*.** HAADF-STEM images of thin-sectioned cells showing a cross-section of the coccolith *in statu nascendi* (blue arrowheads), a cross-section of the coccolith vesicle (CV) – reticular body (RB), a pool of densely concentrated calcium (red arrow), the Golgi apparatus (G), nucleus (N), chloroplast (CP), and mitochondria (M).

**Supplementary Table 1 | Quantities of calcium and phosphorus in calcium-rich bodies calculated from the EDX data set of five cells**

| | Element | Weight % | Atomic % | Uncertainty % | P/Ca ratio |
|---|---|---|---|---|---|
| Cell 1 | P(K) | 63.17 | 66.3 | 1.06 | 2.76 |
| | Ca(K) | 29.54 | 23.96 | 0.71 | |
| Cell 2 | P(K) | 31.60 | 29.18 | 1.03 | 1.83 |
| | Ca(K) | 22.37 | 15.97 | 0.84 | |
| Cell 3 | P(K) | 59.46 | 62.3 | 1.39 | 2.65 |
| | Ca(K) | 29.07 | 23.53 | 0.92 | |
| Cell 4 | P(K) | 60.08 | 59.88 | 1.63 | 3.49 |
| | Ca(K) | 22.26 | 17.15 | 1.06 | |
| Cell 5 | P(K) | 53.44 | 55.76 | 0.67 | 2.22 |
| | Ca(K) | 31.20 | 25.16 | 0.46 | |

The quantities of calcium and phosphorus were determined from the equation: $C_A/C_B = K_{AB} * (I_A/I_B)$ where C stands for the concentration and I for the intensity of the components A and B, using the Tecnai imaging and analysis software TAI.
